# Phosphodiesterase inhibition and Gucy2C activation enhance tyrosine hydroxylase Ser40 phosphorylation and improve 6-hydroxydopamine-induced motor deficits

**DOI:** 10.1186/s13578-024-01312-7

**Published:** 2024-10-25

**Authors:** Erik H. Douma, Jesse Stoop, Matthijs V. R. Lingl, Marten P. Smidt, Lars P. van der Heide

**Affiliations:** 1 Macrobian-Biotech B.V., Science Park 904, 1098 XH Amsterdam, The Netherlands; 2 Swammerdam Institute for Life Sciences, University of Amsterdam, Room C3.104, Science Park 904, 1098 XH Amsterdam, The Netherlands; 3 Parkinnova Therapeutics B.V., Science Park 904, 1098 XH Amsterdam, The Netherlands

**Keywords:** Parkinson’s disease, Dopamine, TH, Ser40, PDE inhibition, GUCY2C, CAMP, CGMP, 6-OHDA, PITX3

## Abstract

**Background:**

Parkinson’s disease is characterized by a progressive loss of dopaminergic neurons in the nigrostriatal pathway, leading to dopamine deficiency and motor impairments. Current treatments, such as L-DOPA, provide symptomatic relief but result in off-target effects and diminished efficacy over time. This study explores an alternative approach by investigating the activation of tyrosine hydroxylase, the rate-limiting enzyme in dopamine synthesis. Specifically, we explore the effects of phosphodiesterase (PDE) inhibition and guanylate cyclase-C (GUCY2C) activation on tyrosine hydroxylase Ser40 phosphorylation and their impact on motor behavior in a 6-hydroxydopamine (6-OHDA) Parkinson's disease model.

**Results:**

Our findings demonstrate that increasing cyclic nucleotide levels through PDE inhibition and GUCY2C activation significantly enhances tyrosine hydroxylase Ser40 phosphorylation. In a Pitx3-deficient mouse model, which mimics the loss of dopaminergic neurons seen in Parkinson’s disease, Ser40 phosphorylation remained manipulable despite reduced tyrosine hydroxylase protein levels. Moreover, we observed no evidence of tyrosine hydroxylase degradation due to Ser40 phosphorylation, challenging previous reports. Furthermore, both PDE inhibition and GUCY2C activation resulted in improved motor behavior in the 6-OHDA Parkinson’s disease mouse model, highlighting the potential therapeutic benefits of these approaches.

**Conclusions:**

This study underscores the therapeutic potential of enhancing tyrosine hydroxylase Ser40 phosphorylation to improve motor function in Parkinson’s disease. Both PDE inhibition and GUCY2C activation represent promising non-invasive strategies to modulate endogenous dopamine biosynthesis and address motor deficits. These findings suggest that targeting cyclic nucleotide pathways could lead to novel therapeutic approaches, either as standalone treatments or in combination with existing therapies like L-DOPA, aiming to provide more durable symptom relief and potentially mitigate neurodegeneration in Parkinson's disease.

**Supplementary Information:**

The online version contains supplementary material available at 10.1186/s13578-024-01312-7.

## Introduction

Parkinson’s disease, the second most common neurodegenerative disorder [1], is characterized by debilitating motor symptoms primarily arising from a severe deficiency of striatal dopamine. This deficiency results from the progressive degeneration of dopaminergic neurons within the nigrostriatal dopamine pathway [2]. Originating in the midbrain with somatodendritic compartments located in the substantia nigra, these neurons project their dopaminergic terminals to the (dorsal) striatum [3–5]. Current therapies for Parkinson’s disease focus on relieving motor symptoms through dopamine replacement strategies, compensating for impaired dopaminergic neurotransmission [6–8]. Unfortunately, these treatments offer no long-term solutions and are accompanied by inescapable side effects within a few years [9–13]. Consequently, there is a pressing need for innovative strategies in Parkinson’s disease treatment.

One promising avenue involves exploring the intricate mechanisms underlying dopamine biosynthesis [14]. Tyrosine hydroxylase, a pivotal enzyme in dopamine synthesis, has drawn particular attention [15]. It converts L-tyrosine into L-3,4-dihydroxyphenylalanine (L-DOPA), the direct precursor of dopamine, and plays a rate-limiting role in dopamine production [3, 16–18]. The enzyme’s activity is tightly regulated by the phosphorylation of its serine 40 (Ser40), which serves to release catecholaminergic feedback inhibition [19–22]. Given that catecholamines exert strict inhibitory control over tyrosine hydroxylase, the enzyme typically remains in a low-activity state with minimal Ser40 phosphorylation [23, 24]. Therefore, Ser40 phosphorylation acts as a crucial gatekeeping mechanism, effectively functioning as an ‘on–off switch’ that governs the activation of tyrosine hydroxylase and, by extension, dopamine production. By promoting Ser40 phosphorylation and thereby initiating its activation, it becomes possible to boost the entire endogenous dopamine biosynthesis pathway [25]. This regulatory mechanism holds significant clinical promise for addressing dopamine deficiency disorders, such as Parkinson’s disease [14].

The cyclic nucleotide second messengers cyclic adenosine monophosphate (cAMP) and cyclic guanosine monophosphate (cGMP) offer powerful means of modulating tyrosine hydroxylase Ser40 phosphorylation. The impact of the cyclic nucleotide second messenger system on Ser40 phosphorylation has been extensively documented [15, 21, 26–33]. Cyclic nucleotide signaling can mediate Ser40 phosphorylation through the activation of various protein kinases, with protein kinase A (PKA) being the most frequently described kinase, validated in multiple experimental contexts [15, 19–22, 26–33]. Fluctuations in cyclic nucleotide second messenger levels significantly impact signaling cascades that, in turn, influence the phosphorylation status of tyrosine hydroxylase [21, 26–33]. Consequently, enhancing cyclic nucleotide signaling emerges as a promising strategy for boosting dopamine biosynthesis.

Two primary mechanisms regulate intracellular cyclic nucleotide levels: (1) their degradation by cyclic nucleotide phosphodiesterases (PDEs) and (2) their production by adenylyl cyclases (ACs) and guanylyl cyclases (GCs) [34–37]. PDEs, which catalyze the hydrolysis of cAMP and cGMP [38–46], present highly promising druggable targets, constituting a diverse and extensive superfamily [34, 37, 38, 45–49]. With their unique catalytic functions, diverse subtypes, and specific tissue and subcellular expression profiles, PDEs emerge as highly promising therapeutic targets for various diseases, particularly those within the central nervous system and those involving critical dopamine signaling pathways [39, 44, 47, 49–54]. Early reports have already demonstrated that inhibiting PDE activity can lead to an upregulation of central mechanisms involved in dopamine synthesis and signaling, including the enzymatic activity of tyrosine hydroxylase [55–59].

In addition to inhibiting PDE activity, the activation of ACs or GCs represents another avenue for increasing intracellular cyclic nucleotide levels. Like PDEs, ACs and GCs exist in multiple isoforms, each with distinct properties and expression profiles across different cell types [35]. Among the GCs, the membrane-bound guanylate cyclase-C (GUCY2C) receptor has emerged as an intriguing target. GUCY2C is a member of the particulate guanylyl cyclases that can be targeted with highly conserved endogenous ligands such as guanylin and uroguanylin. Upon activation, GUCY2C catalyzes the conversion of guanosine triphosphate (GTP) to cGMP [35]. Interestingly, GUCY2C is enriched in midbrain dopaminergic neurons [60–63], making it a promising target for promoting cyclic nucleotide signaling in Parkinson’s disease. Both GUCY2C activation and PDE inhibition offer individual strategies to modulate cyclic nucleotide levels, thereby enhancing dopamine biosynthesis.

In this study, we sought to explore cyclic nucleotide-mediated tyrosine hydroxylase Ser40 phosphorylation as feasible therapeutic approach for addressing the motor symptoms in Parkinson’s disease. First of all, using an ex vivo approach, we investigated tyrosine hydroxylase levels in mouse brain tissue to provide a deeper understanding of the signaling pathways that could be targeted to enhance Ser40 phosphorylation. Through ex vivo striatal brain slice experiments, we confirmed the well-established role of cyclic nucleotide signaling in regulating tyrosine hydroxylase Ser40 phosphorylation. We also examined total tyrosine hydroxylase levels and Ser40 phosphorylation in mice deficient for the transcription factor Pitx3, which serve as a model for dopamine deficiency. Mice with a homozygous deletion of Pitx3 exhibit a significant loss of midbrain dopamine neurons and reduced striatal dopamine innervation, resembling a Parkinson’s-like condition. While these mice exhibited a proportional decrease in tyrosine hydroxylase protein levels, relative Ser40 phosphorylation remained unaffected and responsive to cyclic nucleotide modulation.

Lastly, we investigated the feasibility of enhancing tyrosine hydroxylase Ser40 phosphorylation by inhibiting cyclic nucleotide degradation through PDE2A inhibition and promoting cyclic nucleotide production via Gucy2C activation. Both strategies significantly increased tyrosine hydroxylase Ser40 phosphorylation, underscoring the roles of PDE2A and GUCY2C in dopamine biosynthesis. Furthermore, both PDE2A inhibition and GUCY2C activation ameliorated motor impairments in a Parkinson’s disease mouse model with partial 6-OHDA lesions. These findings highlight the therapeutic potential of targeting cyclic nucleotide signaling to enhance tyrosine hydroxylase activity, which could offer a novel treatment strategy for the motor symptom control in Parkinson’s disease.

## Materials & methods

### Chemicals

IBMX (2845) and PF05180999 (6405) were purchased from ToCris, forskolin (3828) and pCPT-cAMP (C3912) from Cell Signaling Technology, BAY 60-7550 (sc-396772) and guanylin (sc-203067) from Santa Cruz Biotechnology, and uroguanlyin (PUG-4354-S) from Biosynth. Except for guanylin and uroguanylin (demineralized water), all chemicals are dissolved in dimethyl sulfoxide (DMSO; 317275, Merck).

### MN9D cell culture

The dopaminergic MN9D cell line [64] was a kind gift of Dr. Thomas Perlmann [65] and cultured in high glucose Dulbecco’s Modified Eagle’s Medium (DMEM; D6429, Sigma-Aldrich) supplemented with 2 mM L-glutamine (25030081, ThermoFisher Scientific), 1 unit/ml Penicillin/Streptomycin (15140163, ThermoFisher Scientific), and 10% (v/v) heat inactivated fetal bovine serum (HiFBS; S181B, Biowest). The cells are grown and maintained on poly-d-lysine (PDL)-coated 10 cm dishes and cultured in a 37 °C incubator with a humidified atmosphere of 95% air and 5% CO_2_. For passaging, cells were not allowed to exceed 90% confluency and were not used above passage 30. Cultures were rinsed with phosphate buffered saline (PBS; 10010023, ThermoFisher Scientific) and incubated with 1 mL 1X trypsin (15400054, ThermoFisher Scientific) in PBS (10010023, ThermoFisher Scientific) for 5 min. For experiments where protein levels were determined, cells were seeded in PDL-coated 24-well plates. For the fluorescent live cell imaging experiments, see section below.

### MN9D transient transfections for Flamindo2 overexpression and fluorescent live cell imaging

MN9D cells were cultured and maintained as described above. For experiments, cells were seeded in 6-well plates onto PDL-coated 24-mm coverslips. For transient transfections of these MN9D cells, cells were grown in serum-rich DMEM which is replaced to serum-free DMEM prior to the transfection. A mixture of DNA plasmid encoding Flamindo2 and lipofectamine 3000 reagent (L3000001, Invitrogen) was prepared according to manufacturer’s instructions and incubated with the cells. The plasmid encoding Flamindo2 (73938, Addgene) was purchased by the University of Amsterdam’s Molecular Cytology group of Dr. Ir. Joachim Goedhart. After ~ 8 h, the serum-free DMEM was replaced for fresh serum-rich DMEM and incubated for another ~ 36–48 h till fluorescent live cell imaging.

Fluorescent live cell imaging was performed in 750 µL microscopy medium (20 mM HEPES pH 7.4; 137 mM NaCl; 5.4 mM KCl; 1.8 mM CaCl_2_; 0.8 mM MgSO_4_; 20 mM d-Glucose) in a 37 °C incubation chamber using a Zeiss Anxiovert 200 M (semi-)widefield microscope with a Cairn Xenon Arc lamp, in combination with a computer controlled monochromator (bandwidth 0.30 nm), and a 40 × Zeiss Plan-Neofluor oil-immersive objective lens. In addition, a 488–512 nm excitation band-pass (BP) filter and 535-30 YFP BP camera emission filter were used for measuring fluorescent intensity of cells transfected with Flamindo2. Images were collected every 20 s using Metamorph 6.1 software. Images were analyzed using ImageJ software (National Institutes of Health, USA). Collected data was standardized over the baseline period (i.e. first 100 s), normalized to timepoint 0 (i.e. the moment the cells were treated with one of the compounds or vehicle [DMSO for forskolin and BAY 60-7550, microscopy medium for PF05180999]), and presented as a 95% confidence interval showing the change in fluorescence intensity relative to the baseline period (∆F/F). To correct for the photobleaching effect, we calculated a 95% confidence interval of the difference between the experimental compound and the vehicle condition. In this case, data is represented as percentual change in fluorescence intensity of the experimental compound as compared to vehicle condition. Statistical significance was determined by the 95% confidence interval. When at a specific time-point the 95% confidence intervals do not overlap (uncorrected conditions) or when the 95% confidence interval does not overlap with the 0% baseline (photobleaching-corrected condition), the responses in fluorescence intensity that are induced by forskolin, BAY 60-7550, or PF0518099 will be considered significantly different from the vehicle condition.

### In vitro chemical treatment and sample preparation

In vitro experiments were executed in ~ 16 h serum-deprived DMEM (0.5% HiFBS) in order to limit growth-factor interference. All reactions were carried out at 37 °C in the same incubator as used for culturing. Control conditions were all treated with the appropriate amount of vehicle. After treatment, cells were washed with 1X PBS pH 7.4 and lysed in 1X Laemmli sample buffer (62.5 mM Tris–HCl pH 6.8, 2% SDS; [Merck Millipore], 10% glycerol [Sigma-Aldrich] and 0.01% w/v bromophenol blue [Sigma-Aldrich] in MilliQ water supplemented with 50 mM dithiothreitol (DTT; [Merck Millipore]). Samples were collected, sonicated for 3 min in a Bioruptor sonicator (Diagenode) at maximum potency, boiled at 95 °C for 5–10 min, and briefly spun down.

### Animals

All in vivo experiments were performed on adult (~ 3 months old) C57/Bl6/J wild-type or *Pitx3*-deficient mice. The *Pitx3*-deficient mice are either heterozygous for wild-type Pitx3 and green fluorescent protein (GFP) that is knocked-in on the Pitx3 locus (Pitx3^GFP/+^) or homozygous for GFP on the Pitx3 locus (Pitx3^GFP/GFP^). The heterozygous animals are described to have a normal development of the midbrain dopaminergic system, while the homozygous animals are known to have a dramatic loss of neurons of the substantia nigra and its projections to the dorsal striatum [66, 67]. Animals were housed on a 12 h light–dark cycle, with food and water provided ad libitum. All in vivo experiments were conducted according to the European and national legislation.

### Ex vivo slicing and chemical treatment

Mice were sacrificed by cervical dislocation. Brains were immediately isolated and sliced on a Leica VT100S vibratome in ice-cold slicing buffer (120 mM Choline Chloride, 3.5 mM KCl, 0.5 mM CaCl_2_, 6 mM MgSO_4_, 1.25 mM NaH_2_PO_4_, 27.5 mM D-Glucose, 25 mM NaHCO_3_) under constant oxygenation (95% O_2_, 5% CO_2_). Coronal corpus striatum (CS) slices with a thickness of 250 µm were collected over the rostral (R) to caudal (Cd) axis. Subsequently, the slices were micro-dissected and divided in two hemispheres to have an internal control. After micro-dissecting the brain areas of interest, the slices were transferred to 32 °C constantly oxygenized (95% O_2_, 5% CO_2_) artificial cerebrospinal fluid (aCSF; 120 mM NaCl, 3.5 mM KCl, 2.5 mM CaCl_2_, 1.3 mM MgSO_4_, 1.25 mM NaH_2_PO_4_, 27.5 mM D-Glucose, 25 mM NaHCO_3_) for 30 min. Subsequently, the slices were put at room temperature (RT) for another 30 min. In Eppendorf tubes, slices were incubated with—in each experiment specified—compounds that are further diluted in RT aCSF. After pharmacological treatment, aCSF was removed and slices were lysed in warm 1X Laemmli sample buffer (62.5 mM Tris–HCl pH 6.8, 2% SDS; [Merck Millipore], 10% glycerol [Sigma-Aldrich] and 0.01% w/v bromophenol blue [Sigma-Aldrich] in MilliQ supplemented with 50 mM dithiothreitol (DTT; [Merck Millipore]). Samples were sonicated for 3 min in a Bioruptor sonicator (Diagenode) at maximum potency, boiled at 95 °C for 5–10 min, and spun down briefly.

### 6-OHDA surgical procedure and apomorphine rotations test

6-OHDA experiments were performed by Creative Biolabs. Adult male C57/Bl6/J mice were anesthetized with an intramuscular (IM) injection of Zoletil-50S (50 mg/kg) and xylazine hydrochloride (5 mg/kg) and then secured in a stereotaxic frame. A unilateral 6-hydroxydopamine (6-OHDA) lesion was induced by injecting 2 μL of 6-OHDA solution (5 mg/mL) in 0.9% sterile saline, which also contained 0.02% L-ascorbic acid, into the left striatum. The coordinates for the lesion were as follows: Anteroposterior (AP): 1.2 mm; Mediolateral (ML): − 1.1 mm; Dorsoventral (DV): − 4 mm from bregma. The infusion rate was 0.5 µL/min, resulting in a total injection of 5 µg per animal. Sham 6-OHDA-lesioned animals received an identical volume of vehicle alone. Fifteen minutes prior to anesthesia, all animals were pre-treated with a mixture of desipramine hydrochloride (25 mg/kg) and pargyline hydrochloride (5 mg/kg) in 0.9% sterile saline solution (pH 7.4). This pre-treatment was intended to block the uptake of 6-OHDA into norepinephrine-containing terminals and extend the action of 6-OHDA [68]. Concurrently with the 6-OHDA lesion, all mice were implanted with an ICV cannula, which was secured using dental cement. The ICV cannula was targeted to the same side as the lesion site and placed at the following coordinates: AP − 0.26 mm, ML -1.0 mm, DV − 2.8 mm.

Seven days after the 6-OHDA injection, the severity of the lesions was assessed by measuring the rotating behavioral response to apomorphine (0.56 mg/kg, s.c). Successful dopamine deafferentation was defined as animals making at least 90 but fewer than 210 contralateral turns to the side of the 6-OHDA lesion within 30 min of apomorphine injection. From day seven to day 28, animals received daily treatments. Sham 6-OHDA-lesioned animals (*n* = 8) received a daily vehicle injection. In contrast, 6-OHDA-lesioned animals received daily injections of either vehicle (*n* = 12), L-DOPA (*n* = 12; 20 mg/kg, i.p.), PF05180999 (*n* = 12; 30 mg/kg, i.p.), or guanylin (*n* = 12; 0.53 µg, icv). On day 21 and day 28, additional apomorphine rotations tests were conducted to assess the in vivo efficacy of the different treatments. Data are expressed as net contralateral turns for the apomorphine test.

### Capillary-based western blot analysis

Evaluation of protein expression was performed using the Wes™ automated capillary western blot system (Protein Simple, San Jose, CA, USA) according to manufactures instructions and under the default settings. Briefly, prepared cell lysate samples were diluted with MilliQ water, combined with the fluorescent master mix (PS-ST01EZ-8, ProteinSimple), and heated at 95 °C for 5 min. The samples, biotinylated ladder (PS-ST01EZ-8, ProteinSimple), reagents (including the secondary antibody) from the anti-rabbit detection module (DM-001, ProteinSimple), and primary antibodies were loaded into designated wells in the 12–230 kDa separation module assay plate (PS-PP03, ProteinSimple). The assay plate and a 25-capillary cartridge are inserted into the Wes™ machine. The machine automatically separates the proteins by size and performs the immunoprobing, incubations, washing steps, and detection. Digital images were analyzed using the Compass software (ProteinSimple). Proteins were probed using the following antibodies: rabbit anti-Tyrosine Hydroxylase (P40101, Pel-Freez); rabbit anti-phospho-tyrosine hydroxylase (Ser40) (2791S, CST); rabbit anti-β-actin (4907S, CST). Antibodies were diluted in antibody diluent (1:10 [mouse striatum; 2791S, CST] or 1:50 [all others]; 042–203, ProteinSimple).

### Statistical analysis

The amount of phospho-protein levels are corrected for the total amount of the same protein, phosphorylated or not, and normalized to the vehicle condition. For the in vitro experiments, one way analysis of variance (ANOVA) was utilized and followed by Bonferroni’s multiple comparisons post hoc testing to determine statistical significance. To determine statistical significance for the mouse ex vivo experiments comparing two groups, two-tailed paired student’s *t*-tests were used. For the ex vivo experiments comparing the Pitx3 animals and to examine the effects of Ser40 phosphorylation on overall tyrosine hydroxylase levels, area under the curve (AUC) values were determined for each condition per animal for which outer values (the most rostral and most caudal slices) were determined by interpolation. Subsequently, significance was determined by two-tailed paired student’s *t*-tests. The data are expressed as fold change relative to the control condition (control = 1) and presented as bar charts (showing the mean ± SEM; *n* ≥ 3) or minimum to maximum boxplots (showing the first quartile, third quartile, and the mean). The in vivo apomorphine-induced rotational behavior data were analyzed using one-way analysis of variance (ANOVA) followed by Bonferroni’s post hoc test for multiple comparisons to assess the effects of different treatments on motor behavior across time points. Rotational behavior was expressed as net contralateral turns, and treatment efficacy was evaluated by comparing rotational scores at baseline (pre-treatment) with those at days 21 and 28. Differences were considered to be significant at a *p*-value < 0.05. Asterisks indicate significance (*p < 0.05 and **p < 0.01). For the fluorescent live cell imaging experiments, see "MN9D transient transfections for Flamindo2 overexpression and fluorescent live cell imaging" section.

## Results

### Investigating tyrosine hydroxylase phosphorylation in acute brain slices

We set up an ex vivo pharmacological approach using acute brain slices to precisely investigate tyrosine hydroxylase phosphorylation within specific brain areas. Traditionally, two techniques are used to study tyrosine hydroxylase levels in mouse brain tissue [31]. The first method involves coronal slicing of non-frozen tissue using a brain matrix, followed by freehand or punch dissection of the nuclei of interest [24, 69–77]. Although straightforward, this technique has a high risk of contamination with unwanted structures [31]. The second technique utilizes frozen brain tissue and requires the collection of specific nuclei using a punch [78], necessitating multiple brain slices for a specific brain area but with reduced contamination. Our goal is to manipulate intracellular signaling within a physiologically realistic environment while minimizing contamination from adjacent brain structures. Therefore, we introduced an alternative approach, that allows isolation and investigation of the target brain regions with a high degree of precision, ensuring that the samples primarily consist of the cells and structures of interest.

**Fig. 1:**
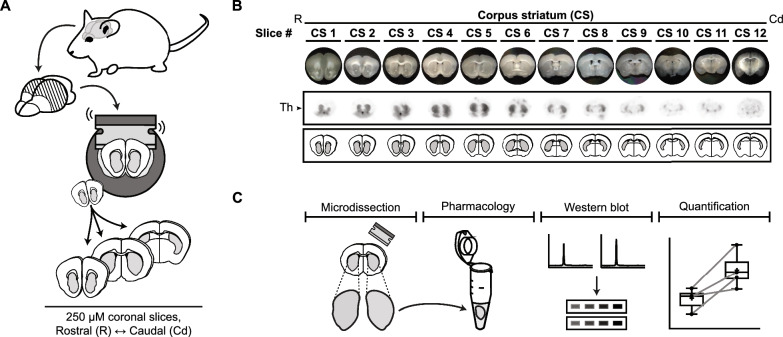
Ex vivo pharmacology approach to determine tyrosine hydroxylase phosphorylation in mouse brain slices. **A** Preparation of mouse coronal slices along the rostral (R) to caudal (Cd) axis. Brains from adult mice were isolated and vibratome-sectioned into 250 µm coronal slices for tissue collection. **B** Overview of the mouse coronal slices along the rostral-caudal axis that contain dopaminergic terminals in the (dorsal) corpus striatum (CS). Light microscopy pictures of each slice are shown together with corresponding in situ blots [173], demonstrating tyrosine hydroxylase (Th) expression within each brain slice. Below the in situ blots are schematic representations of the regions of interest per slice (in gray) that include the tyrosine hydroxylase-containing dopaminergic terminals. **C** Regions of interest are micro-dissected from the collected coronal slices and transferred into Eppendorf tubes for ex vivo pharmacological treatment. In general, one of the two corresponding micro-dissected regions of interest (randomly chosen) is treated with a target compound, while the micro-dissected region of interest from the corresponding hemisphere is treated with vehicle. Following treatment, protein levels were determined by (automated) western blot analysis. Quantifications are presented as fold change compared to the normalized control condition

In our approach, we prepared and examined living acute brain slices, a method primarily used in electrophysiology [79], which closely resembles the technique used by Sugiyama *and colleagues* [80]. As shown in Fig. 1, we rapidly extracted the brains from adult mice, sliced non-frozen brains into 250 μm coronal sections using a vibrating blade microtome, and collected the acute slices containing our target brain regions (Fig. 1A). We obtained acute slices containing the dorsal corpus striatum (CS) to manipulate tyrosine hydroxylase phosphorylation in nigrostriatal dopaminergic neurons. This region is implicated in the motor symptoms characteristic of Parkinson's disease due to dopamine deficiency [2]. For adult mice, we typically collected 12 slices to encompass the entire dorsal striatum (Fig. 1B).

To minimize contamination from surrounding brain structures, we micro-dissected the regions of interest in each collected slice (depicted in gray in Fig. 1B) freehand under ice-cold (but not freezing) conditions using a light microscope (Fig. 1C). We conducted ex vivo pharmacology experiments on these micro-dissected acute brain slices in polypropylene incubation tubes (Fig. 1C). Finally, we employed the Wes™ system (ProteinSimple) for automated western blot analysis, enabling us to detect and analyze protein levels in each micro-dissected slice (Fig. 1C). Unlike cell cultures or homogenates, acute brain slices maintain their in vivo cytoarchitecture and synaptic circuitry, allowing the isolated study of specific brain regions. Consequently, our approach facilitates the investigation and control of tyrosine hydroxylase phosphorylation in specific brain regions with minimal contamination from adjacent structures, while preserving the optimal cellular, molecular, and circuitry characteristics of the regions of interest [79, 81].

**Fig. 2:**
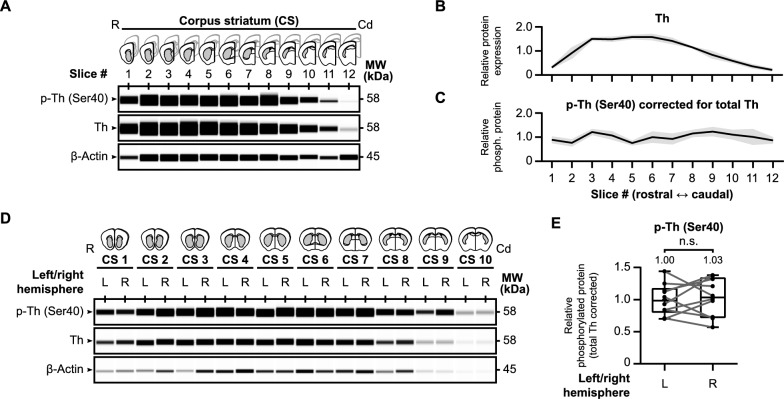
Tyrosine hydroxylase protein distribution in mouse striatal slices. **A** Schematic images of each examined brain slice (*n* = 3) over the rostral-caudal axis of the corpus striatum (CS) with representative western blot images for baseline protein levels of tyrosine hydroxylase phosphorylated at Ser40 (p-Th), overall tyrosine hydroxylase (Th), and β-actin. **B** Quantitative analysis demonstrates a right-skewed peak in tyrosine hydroxylase levels in the more rostral part of the striatum. **C** When Ser40 phosphorylated tyrosine hydroxylase levels are corrected for overall tyrosine hydroxylase levels, a uniform distribution of relative Ser40 phosphorylation levels can be observed along the rostral-caudal axis of the striatum. **D**–**E** There are no differences in relative striatal tyrosine hydroxylase Ser40 phosphorylation levels between the left and right hemisphere. Micro-dissected regions of interest from the left (L) and right (R) hemisphere were carefully separated and examined for baseline (phospho-) tyrosine hydroxylase levels (**D**). Although there is variance between hemispheres (*n* = 10), there is no hemisphere-dependent effect on relative tyrosine hydroxylase Ser40 phosphorylation levels (**E**)

First of all, we assessed tyrosine hydroxylase levels across the rostro-caudal axis of the mouse brain (*n* = 3) as shown in Fig. 2. Each coronal slice, obtained following the procedure described earlier, was divided into hemispheres, and the regions of interest (depicted in gray in schematic images of Fig. 2A) were micro-dissected. We analyzed the micro-dissected parts of each coronal slice for baseline protein levels of total tyrosine hydroxylase, regardless of phosphorylation status, phospho-tyrosine hydroxylase (Ser40), and β-actin (Fig. 2A). The quantification of total tyrosine hydroxylase protein levels revealed a specific pattern along the rostro-caudal axis. There was a right-skewed peak in tyrosine hydroxylase levels in the more rostral part of the striatum (Fig. 2B). Importantly, when we quantified the phospho-tyrosine hydroxylase levels and adjusted them for the total tyrosine hydroxylase levels, a more uniform distribution was observed (Fig. 2C). This indicates even distribution of Ser40 phosphorylation levels in the dorsal striatum along the rostral-caudal axis.

Our primary objective is to manipulate tyrosine hydroxylase phosphorylation within our acute brain slices, focusing on the dopaminergic terminals in the striatum. Our approach involves dividing each slice into hemispheres and micro-dissecting the regions of interest in each slice. For every slice, we employed the micro-dissected part of one hemisphere as an internal control for the experimental, treated condition, which is the micro-dissected part of the other hemisphere. While the control and experimental conditions are chosen at random, this setup will only be suitable if the relative tyrosine hydroxylase levels are similar between hemispheres. Therefore, we carefully segregated the slices from the left hemisphere from those in the right hemisphere and analyzed protein levels of total tyrosine hydroxylase and phospho-Ser40 tyrosine hydroxylase (Fig. 2D). In summary, we found no significant differences in relative Ser40 phosphorylation levels between the left and right hemisphere (Fig. 2E, t(9) = 0.38, *p* > 0.05, *M*_Left_ = 1.00, *M*_Right_ = 1.03). As such, the relative phosphorylation levels remained not only consistent throughout the striatum, but as well between hemispheres. In conclusion, our approach enables the investigation and manipulation of tyrosine hydroxylase in non-frozen micro-dissected brain tissue, offering a valuable tool for future studies.

### Mouse striatal tyrosine hydroxylase phosphorylation is regulated via the cyclic AMP second messenger system

**Fig. 3:**
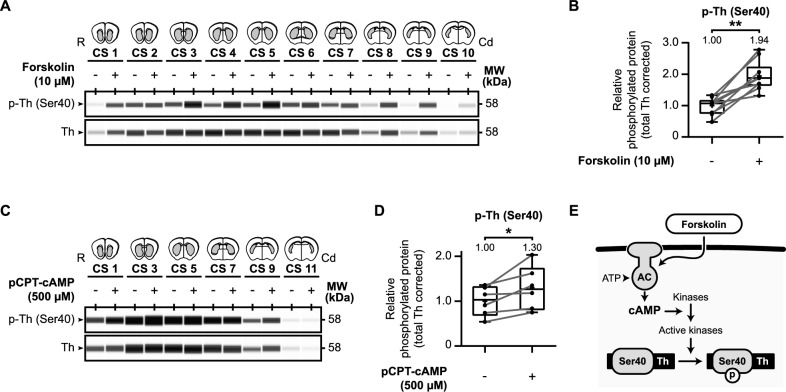
Cyclic nucleotide-mediated signaling is involved in tyrosine hydroxylase Ser40 phosphorylation in the mouse striatum. **A** Forskolin upregulates tyrosine hydroxylase Ser40 phosphorylation in the mouse striatum. Micro-dissected mouse striatal slices (*n* = 10) were exposed for 60 min to either vehicle or 10 µM forskolin and examined for (phospho-) tyrosine hydroxylase levels. **B** Quantitative analysis of relative Ser40 phosphorylation shows that exposure to forskolin induces relative Ser40 phosphorylation. **C** The cAMP analogue pCPT-cAMP upregulates tyrosine hydroxylase Ser40 phosphorylation in the mouse striatum. Micro-dissected mouse striatal slices (*n* = 6) were exposed for 60 min to either vehicle or 500 µM pCPT-cAMP and examined for (phospho-) tyrosine hydroxylase levels. **D** Quantitative analysis of relative Ser40 phosphorylation shows that exposure to pCPT-cAMP induces relative Ser40 phosphorylation. **E** Forskolin is an adenylyl cyclase (AC) activator that increases intracellular levels of the second messenger cyclic adenosine monophosphate (cAMP). Activation of AC via forskolin stimulates the catalyzation of adenosine triphosphate (ATP) to cAMP, leading to increased activation of specific kinases that can phosphorylate (p) tyrosine hydroxylase (Th) at Ser40

A wealth of data described that tyrosine hydroxylase is affected by the cAMP second messenger system [21, 26–31]. Therefore, we investigated the influence of cAMP signaling on tyrosine hydroxylase phosphorylation using our ex vivo approach (Fig. 3), either via forskolin (Fig. 3A, B) or via pCPT-cAMP (Fig. 3C, D), a cell-permeable analogue of cAMP.

Forskolin is used to increase cAMP levels via the activation of adenylyl cyclases (ACs) [82] and is known to affect tyrosine hydroxylase phosphorylation [83, 84]. We collected and micro-dissected coronal striatal slices as described earlier. Per collected slice, one micro-dissected region of interest was exposed to 10 µM forskolin for 60 min while the matching part was treated with DMSO vehicle. Subsequently, tyrosine hydroxylase protein levels were examined (Fig. 3A, B). Relative Ser40 phosphorylation levels are significantly increased due to forskolin exposure (Fig. 3B; *t*(9) = 6.48, *p* < 0.01, *M*_Forskolin_ = 1.94). In a similar approach we tested the effects of 60 min exposure to 500 µM pCPT-cAMP [85–88] on tyrosine hydroxylase phosphorylation (Fig. 3C, D) and demonstrated that pCPT-cAMP elevated relative Ser40 phosphorylation levels (Fig. 3D; *t*(5) = 2.83, *p* < 0.05, *M*_pCPT-cAMP_ = 1.30).

In conclusion, in the mouse striatum Ser40 phosphorylation is upregulated by the activation of the cAMP second messenger system, via both forskolin and the cAMP analogue (Fig. 3E).

### In the Pitx3-deficiency mouse model for selective loss of nigrostriatal dopamine neurons, tyrosine hydroxylase levels are substantially lower, but relative Ser40 phosphorylation levels remain unaffected

From a therapeutic perspective, we aim to manipulate Ser40 phosphorylation in a dopamine deficient setting similar to the situation of Parkinson’s disease patients. To mimic such a situation, we used the Pitx3-deficiency mouse model. Pitx3 is a transcription factor crucial for normal substantia nigra dopamine neuron development [89–92]. Mice deficient in Pitx3 serve as a model for the selective loss of nigrostriatal dopamine neurons and exhibit impaired performance on specific behavioral tests, which is reversible with L-DOPA treatment [92, 93].

To investigate the effects of Pitx3-deficiency on tyrosine hydroxylase levels and regulation in the striatum, we compared tyrosine hydroxylase protein levels between phenotypically normal heterozygous *Pitx3*^GFP/+^ and phenotypically defective homozygous *Pitx3*^GFP/GFP^ mice. The heterozygous mutation of *Pitx3* does not affect nigrostriatal dopamine neuron development and these mice show similar tyrosine hydroxylase levels to *Pitx3* wild-type littermates [66, 67], making these mice suitable controls for the Pitx3-deficient mice.

**Fig. 4:**
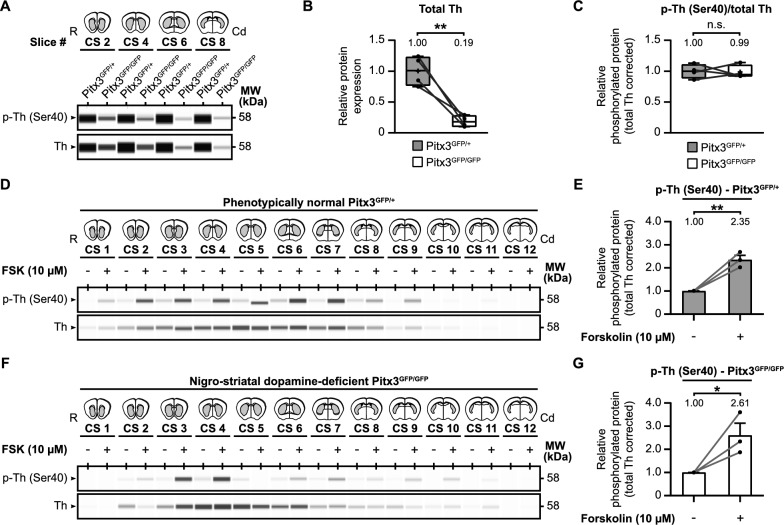
The effect of Pitx3-deficiency on tyrosine hydroxylase levels in the mouse striatum. Deficiency in the homeobox gene *Pitx3* leads to selective neuronal cell loss of the same group of dopamine neurons that are affected in Parkinson’s disease. As a consequence of this selective loss of neurons, dopaminergic connections to the striatum are affected as well. To examine the effects of *Pitx3*-deficiency on tyrosine hydroxylase (Th) levels (**A**–**C**) and regulation (**D**–**G**) in the striatum, we examined and compared total Th and phospho-Th (Ser40) protein levels between phenotypically normal *Pitx3*^GFP/+^ and defective *Pitx3*^GFP/GFP^ mice. **A** Representative western blot images for protein levels of Th and phospho-Th (Ser40) of four untreated micro-dissected striatal mouse brain slices over the rostral (R)-caudal (Cd) axis, comparing striatal Th levels of *Pitx3*^GFP/+^ and *Pitx3*^GFP/GFP^ mice. Per slice, protein levels were averaged over animals (*n* = 3) and the averages of *Pitx3*^GFP/+^ mice were compared with the averages of *Pitx3*^GFP/GFP^ mice. **B** Quantitative analysis of total tyrosine hydroxylase (Th) levels. Tyrosine hydroxylase levels are drastically lower in the phenotypically defective *Pitx3*^GFP/GFP^ mice. **C** Quantitative analysis of relative tyrosine hydroxylase Ser40 phosphorylation levels. Relative Ser40 phosphorylation levels are comparable between the two genotypes. **D**–**G** The effect of *Pitx3*-deficiency on tyrosine hydroxylase regulation by forskolin. Twelve micro-dissected mouse striatal slices of (**D**) *Pitx3*^GFP/+^ mice (*n* = 3) or (**F**) *Pitx3*^GFP/GFP^ (*n* = 3) mice were exposed to either 10 μM forskolin or vehicle for 60 min. Quantitative analysis of relative tyrosine hydroxylase Ser40 phosphorylation levels demonstrates that forskolin upregulates tyrosine hydroxylase Ser40 phosphorylation in both (**E**) *Pitx3*^GFP/+^ mice and (**G**) *Pitx3*^GFP/GFP^ mice, and to a similar extent

Our initial objective was to determine if any compensation occurs at the level of tyrosine hydroxylase phosphorylation in a dopamine-deficient context (Fig. 4A–C). We compared tyrosine hydroxylase levels in the striatum of *Pitx3*^GFP/+^ and *Pitx3*^GFP/GFP^ mice. As expected, tyrosine hydroxylase levels were significantly lower in *Pitx3*^GFP/GFP^ mice (Fig. 4B; *t*(3) = 5.85, *p* < 0.01, *M* = 0.19), reaching approximately one fifth of the levels in *Pitx3*^GFP/+^ mice. However, there was no difference in the relative striatal Ser40 phosphorylation levels between the two genotypes (Fig. 4C; *t*(3) = 0.12, *p* > 0.05, *M* = 0.99).

Next, we examined the potential for manipulating tyrosine hydroxylase phosphorylation in a tyrosine hydroxylase-deficient situation (Fig. 4D–G). We exposed striatal slices from both *Pitx3*^GFP/+^ (Fig. 4D, E) and *Pitx3*^GFP/GFP^ (Fig. 4F, G) mice to forskolin (10 µM) for 60 min. Quantification revealed that both the *Pitx3*^GFP/+^ mice (Fig. 4E; *t*(4) = 7.08, *p* < 0.01, *M*_FSK_ = 2.35) and *Pitx3*^GFP/GFP^ mice (Fig. 4G; *t*(4) = 3.11, *p* < 0.05, *M*_FSK_ = 2.61) exhibited a similar increase in relative Ser40 phosphorylation levels upon forskolin exposure, comparable to the observations in wild-type C57/Bl6/J mice (Fig. 3B).

In summary, the Pitx3-deficiency mouse model represents selective nigrostriatal dopamine neuron loss, accompanied by substantially lower tyrosine hydroxylase levels. Notably, relative Ser40 phosphorylation levels remain unchanged compared to the phenotypically normal situation, and they can still be upregulated by forskolin to a similar extent. This suggests that relative Ser40 phosphorylation levels can be enhanced even in situations where a significant proportion of nigrostriatal dopaminergic neurons are lost, and overall dopamine levels are reduced.

### Pde2A inhibition leads to immediate elevations of cAMP levels in MN9D cells

Subsequently, we investigated the influence of PDE2A inhibition on cyclic nucleotide levels. PDEs, although promising therapeutic targets, are complex subjects due to the precise regulation of intracellular cyclic nucleotide concentrations and their compartmentalized downstream signaling [34, 37]. PDEs are intricately involved in these processes in a cell-specific manner [47, 94] and can perform roles beyond cyclic nucleotide hydrolysis, such as acting as scaffolding proteins or inducing allosteric changes that affect protein–protein interactions [45]. Hence, blocking the activity of a specific PDE does not necessarily lead to increased cyclic nucleotide levels and an expected gain of function, as one might anticipate based solely on their enzymatic function.

**Fig. 5:**
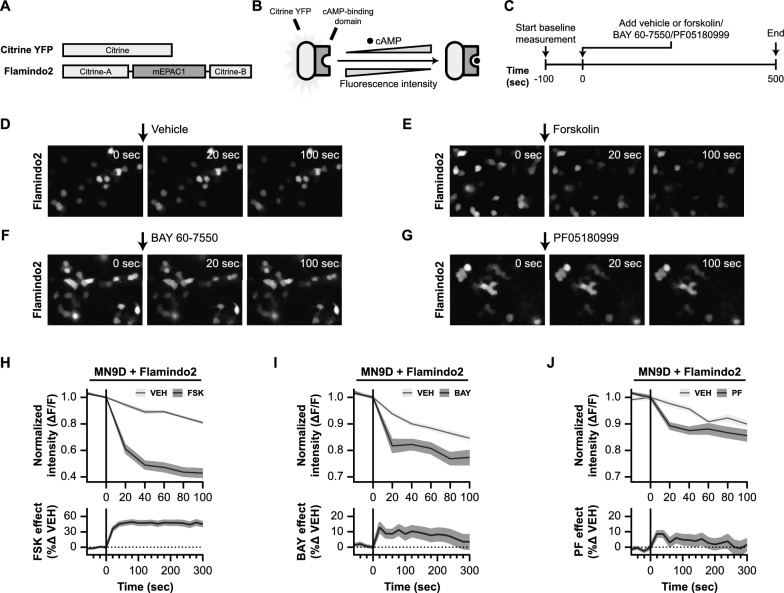
Live cell imaging in Flamindo2-expressing MN9D cells demonstrates that PDE2A inhibition leads to increased levels of cAMP. **A** Schematic representation of the domain structure of Flamindo2. Citrine is a mutant of yellow fluorescent protein (YFP). Flamindo2 is created by insertions of DNA fragments that encode the cAMP binding domain of mEPAC2. **B** Schematic working mechanism of Flamindo2. Binding of cAMP to the cAMP binding domain reduces fluorescence of the Flamindo2 protein. Therefore, decreasing fluorescence indicates elevations in cAMP levels within a cell. **C** Experimental timeline of the live cell imaging experiments in Flamindo2 expressing MN9D cells. Prior to application of vehicle, forskolin, or phosphodiesterase (PDE) inhibitors, a baseline measurement in fluorescence intensity of 100 s was performed. Images were collected every 20 s. **D**–**G** Representative images showing fluorescence intensity just before application of the compounds at 0 s, at 20 s of incubation with the compound, and after 100 s. Representative image are shown for treatment with a vehicle (**D**), 10 µM forskolin (**E**), 10 µM BAY 60-7550 (**F**), and 100 µM PF05180999 (**G**). **H**–**J** 95% confidence intervals time-courses representing the changes in fluorescence intensity induced by forskolin (**H**), BAY 60-7550 (**I**), or PF05180999 (**J**). Upper graphs show change in fluorescence intensity relative to the baseline period (∆F/F), while lower graphs are photobleaching effect-corrected representations of the percentual change in fluorescence intensity of each compound as compared to their vehicle

Therefore, we first examined the effects of PDE2A inhibitors BAY 60-7550 and PF05180999 on cAMP levels (Fig. 5). To do this, we employed the YFP-based cAMP indicator named Flamindo2 [95]. Flamindo2 is a single fluorescent protein (FP)-based intensiometric indicator that allows monitoring intracellular cAMP levels in living cells via changes in fluorescence intensity. In Flamindo2, the YFP variant citrine is fused with a cAMP binding domain of mEPAC1 (Fig. 5A), which results in changes in fluorescence intensity upon binding of cAMP (Fig. 5B). We introduced Flamindo2 to dopaminergic MN9D cells and assessed the effects of forskolin, BAY 60-7550, and PF05180999 on fluorescence intensity (Fig. 5C-J).

First, we tested the effects of forskolin (10 µM), a known adenylyl cyclase activator [82], on the fluorescence intensity of the cAMP reporter protein (Fig. 5E). Forskolin induced a rapid decrease in fluorescence intensity (Fig. 5H; upper graph), signifying an increase in intracellular cAMP levels. The fluorescence intensity of Flamindo2 was decreased by 40% after 20 s, 50% after 40 s, and by 60% after 100 s, without overlapping with the 95% confidence interval of the vehicle condition.

It's worth noting that the vehicle condition also showed a decrease in fluorescence intensity, due to the photobleaching effect of the fluorescent biosensor which progressively decreases the signal-to-noise ratio and is a common limitation of “downward” fluorescence sensors [96, 97]. Yet, this effect is expected in both the vehicle and experimental conditions. When corrected for photobleaching (Fig. 5H; lower graph), forskolin again induced significant and rapid elevations in cAMP levels in MN9D cells.

Next, we examined the effects of the PDE2A inhibitors BAY 60-7550 (Fig. 5F) and PF05180999 (Fig. 5G) on the Flamindo2 cAMP indicator. Similar to the vehicle condition in the forskolin experiment, the vehicle-treated MN9D cells exhibited a decrease in fluorescence intensity over time (Fig. 5I, J; upper graphs). However, BAY 60-7550 (10 µM) and PF0518099 (100 µM) caused more pronounced decreases in fluorescence intensity, which were distinct from their respective vehicle conditions. In summary, our findings suggest that Pde2A inhibition leads to rapid increases in cAMP levels within MN9D cells, although not as potent as forskolin.

### Phosphorylation of tyrosine hydroxylase at Ser40 is upregulated by Pde2A inhibition in MN9D cells

**Fig. 6:**
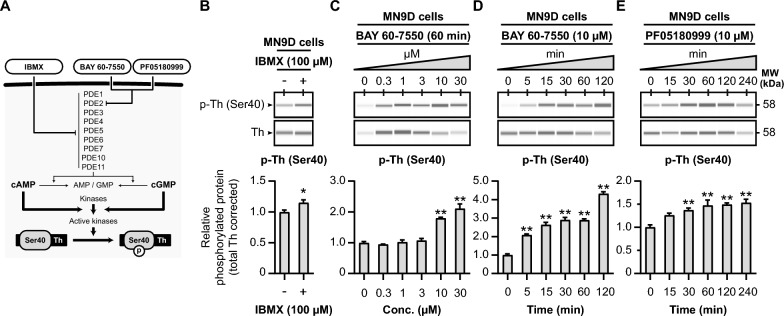
PDE inhibition upregulates tyrosine hydroxylase Ser40 phosphorylation in MN9D cells. **A** Schematic representation of the hypothesized effects of PDE inhibition on tyrosine hydroxylase Ser40 phosphorylation. IBMX is a non-selective PDE inhibitor, inhibiting PDE families PDE1-7, PDE10, and PDE11. The PDE inhibitors BAY 60-7550 and PF05180999 are potent and selective inhibitors to PDE2A. Inhibition of the activity of PDEs will result in less cyclic nucleotide hydrolysis. Therefore, this will lead to more cyclic nucleotide-dependent activation of tyrosine hydroxylase Ser40 phosphorylating kinases. **B** The non-selective PDE inhibitor IBMX (100 µM) increases tyrosine hydroxylase Ser40 phosphorylation levels in dopaminergic MN9D cells (*n* = 4). **C** Dose–response curve of the effects 60 min exposure to the PDE2A inhibitor BAY 60-7550 on tyrosine hydroxylase Ser40 phosphorylation in MN9D cells. Quantitative analysis reveals that BAY 60-7550 upregulates tyrosine hydroxylase Ser40 phosphorylation when at concentrations of 10 µM and 30 µM (*n* = 4). **D** Time-response curve of the effects of 10 µM BAY 60-7550 on tyrosine hydroxylase Ser40 phosphorylation in MN9D cells. Quantitative analysis shows that BAY increases Ser40 phosphorylation at all examined timepoints (*n* = 4). **E** Time-response curve of the effects of 60 min exposure to the PDE2A inhibitor PF05180999 on tyrosine hydroxylase Ser40 phosphorylation in MN9D cells. After 30 min, the PDE2A inhibitor (*n* = 4) was able to significantly upregulate tyrosine hydroxylase Ser40 phosphorylation levels

Earlier, we demonstrated that tyrosine hydroxylase Ser40 phosphorylation is influenced by cyclic nucleotide dynamics (Fig. 3). Having established that the PDE2A inhibitors BAY 60-7550 and PF05180999 can elevate cyclic nucleotide levels in dopaminergic MN9D cells (Fig. 5), we next want to examine whether these changes translate into modifications in tyrosine hydroxylase phosphorylation (Fig. 6).

We begin by exploring the effects of general PDE inhibition using 3-Isobutyl-1-methylxanthine (IBMX), a widely used non-specific PDE inhibitor [39, 98–100] that affects the activity of most PDE families (Fig. 6A), in MN9D cells. The MN9D cell line is a fusion of N18TG2 neuroblastoma cells and mouse dopaminergic midbrain neurons [64], and therefore endogenously expresses tyrosine hydroxylase protein. When MN9D cells are exposed to IBMX (100 µM) for 60 min, we observe an upregulation in relative tyrosine hydroxylase Ser40 phosphorylation levels (Fig. 6B; *p* < 0.05, *M* = 1.15). This demonstrates that PDE inhibition with IBMX can regulate tyrosine hydroxylase phosphorylation in MN9D cells.

Subsequently, we investigate the effects of the PDE2A inhibitors BAY 60-7550 (Fig. 6C, D) and PF05180999 (Fig. 6E) on relative tyrosine hydroxylase Ser40 phosphorylation levels in MN9D cells. First, MN9D cells are treated with different concentrations of BAY 60-7550 (0, 0.3, 1, 3, 10, and 30 µM) for 60 min (Fig. 6C). In this experiment, BAY 60-7550 significantly increases relative Ser40 phosphorylation (F(5, 18) = 42.39, *p* < 0.01), with a notable effect observed at concentrations of 10 and 30 µM (Fig. 6C; *p* < 0.01, *M* > 1.79).

We further assess the time-dependent effects of BAY 60–7550 (10 µM) on tyrosine hydroxylase protein levels in MN9D cells (Fig. 6D). BAY 60–7550 is capable of rapidly increasing relative tyrosine hydroxylase Ser40 phosphorylation levels (F (5, 18) = 116.70, *p* < 0.01), with significant effects as early as 5 min of exposure (Fig. 6D; *p* < 0.01, *M* > 2.09).

Lastly, we expose MN9D cells to the other PDE2A inhibitor, PF05180999 (10 µM), for varying durations (0, 15, 30, 60, 120, and 240 min; Fig. 6E). PF05180999 also leads to an increase in relative Ser40 phosphorylation levels (F (5, 18) = 8.43, *p* < 0.01). While after 15 min no significant effect can be seen (*p* = 0.07, *M* = 1.26), from 30 min and on PF05180999 was able to increase relative Ser40 phosphorylation levels (Fig. 6E; *p* < 0.01, *M* > 1.37).

To summarize, our data demonstrates that PDE inhibition via IBMX, as well as through the PDE2A inhibitors BAY 60-7550 and PF05180999, effectively upregulates tyrosine hydroxylase Ser40 phosphorylation in dopaminergic MN9D cells.

### Mouse striatal tyrosine hydroxylase Ser40 phosphorylation is upregulated by Pde2A inhibition

In the previous section, we demonstrated in dopaminergic MN9D cells that tyrosine hydroxylase Ser40 phosphorylation can be enhanced through general PDE inhibition with IBMX and, more specifically, through PDE2A inhibition using BAY 60-7550 and PF05180999. Now, we aim to explore the impact of inhibiting PDE activity on tyrosine hydroxylase phosphorylation in a more physiologically relevant environment. Consequently, we examined the effects of the previously mentioned PDE inhibitors on tyrosine hydroxylase Ser40 phosphorylation in the mouse striatum.

**Fig. 7:**
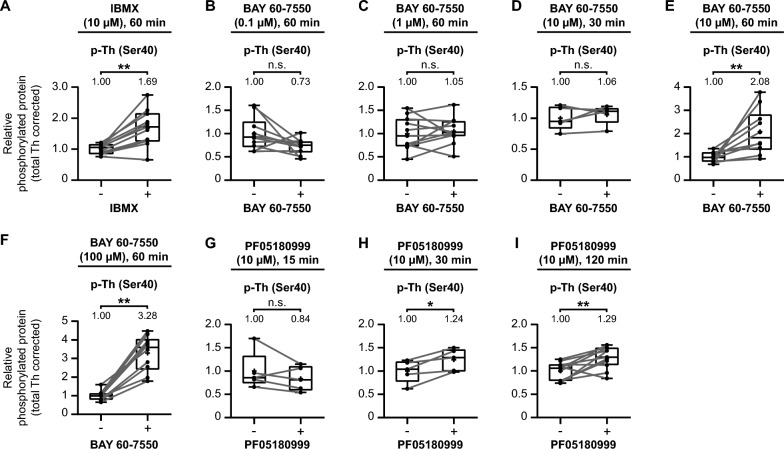
PDE inhibition upregulates tyrosine hydroxylase Ser40 phosphorylation in the mouse striatum. Quantitative analysis of the effects of the non-selective PDE inhibitor IBMX (**A**), different concentrations and/or times of exposure to the PDE2A inhibitor BAY 60-7550 (**B**–**F**), or the PDE2A inhibitor PF05180999 (**G**–**I**) on tyrosine hydroxylase Ser40 phosphorylation in mouse striatal slices. **A** Exposure to the non-selective PDE inhibitor IBMX (10 µM) for 60 min upregulates tyrosine hydroxylase Ser40 phosphorylation in mouse striatal slices (*n* = 11). **B–F** Exposure to the PDE2A inhibitor BAY 60-7550 for 60 min at concentrations of 0.1 µM (**B**; *n* = 10) and 1 µM (**C**; *n* = 11) have no effect on Ser40 phosphorylation, as well as after 30 min at a concentration of 10 µM (**D**; *n* = 5). However, after 60 min exposure at concentrations of 10 µM (**E**; *n* = 10) and 100 µM (**F**; *n* = 10), BAY 60-7550 upregulates tyrosine hydroxylase Ser40 phosphorylation in mouse striatal slices. **G**–**I** Exposure to the PDE2A inhibitor PF05180999 (10 µM) for 15 min had no effect (**G**; *n* = 5) on tyrosine hydroxylase Ser40 phosphorylation in mouse striatal slices, while it upregulates Ser40 phosphorylation after both 30 (**H**; *n* = 5) and 120 (**I**; *n* = 11) minutes of exposure to PF05180999

We employed the same methodology as described earlier: collecting and micro-dissecting coronal striatal mouse brain tissue (Fig. 1). In each specific experiment, one micro-dissected region of interest per collected slice was exposed to IBMX (Fig. 7A), BAY 60–7550 (Fig. 7B–F), or PF05180999 (Fig. 7G-I), while the corresponding part was treated with vehicle. See Supplementary Figure S1 for the blot-like images of each individual experiment.

The effects in the mouse striatum mirrored those observed in dopaminergic MN9D cells. Firstly, the non-selective PDE inhibitor IBMX (10 µM) significantly elevated tyrosine hydroxylase Ser40 phosphorylation levels (Fig. 7A; *t*(10) = 4.99, *p* < 0.01, *M* = 1.69).

Next, we investigated the effects of the PDE2A inhibitor BAY 60–7550 on tyrosine hydroxylase phosphorylation. Mouse striatal slices exposed to BAY 60–7550 for 60 min at concentrations of 0.1 µM (Fig. 7B; *t*(9) = 2.01, *p* = 0.08, *M* = 0.73) or 1 µM (Fig. 7C; *t*(10) = 0.57, *p* = 0.58, *M* = 1.05) showed no significant differences compared to the vehicle conditions. However, at a concentration of 10 µM, there was no significant effect after 30 min (Fig. 7D; *t*(4) = 0.84, *p* = 0.45, *M* = 1.06), but a significant increase was observed after 60 min (Fig. 7E; *t*(9) = 3.43, *p* < 0.01, *M* = 2.08). Further increasing the concentration to 100 µM resulted in an even more potent increase in Ser40 phosphorylation levels (Fig. 7F; *t(*9) = 8.57, *p* < 0.01, *M* = 3.28).

Finally, we assessed the effects of PF05180999 (10 µM) on tyrosine hydroxylase Ser40 phosphorylation. PF05180999 had no significant effect on tyrosine hydroxylase Ser40 phosphorylation after 15 min (Fig. 7G; *t(*4) = 1.36, *p* = 0.25, *M* = 0.84). However, at 30 min (Fig. 7H; *t(*4) = 3.82, *p* < 0.05, *M* = 1.24) and 120 min (Fig. 7I; *t(*10) = 3.53, *p* < 0.01, *M* = 1.29) of PDE2A inhibition with PF05180999, we observed increased levels of tyrosine hydroxylase Ser40 phosphorylation in the mouse striatum.

In summary, similar to MN9D dopaminergic cells, both non-specific PDE inhibition via IBMX and Pde2A inhibition through BAY 60-7550 and PF05180999 can upregulate tyrosine hydroxylase Ser40 phosphorylation in the mouse striatum. These effects are concentration- and time-dependent.

### Mouse striatal tyrosine hydroxylase Ser40 phosphorylation is upregulated by Gucy2C activation

In the previous section, we demonstrated that tyrosine hydroxylase Ser40 phosphorylation could be enhanced through PDE inhibition in the mouse striatum. To further explore cyclic nucleotide signaling mechanisms, we investigated whether GUCY2C activation could similarly upregulate tyrosine hydroxylase Ser40 phosphorylation. Given that cGMP can activate protein kinases involved in the phosphorylation of tyrosine hydroxylase at Ser40 [21], we hypothesized that stimulating Gucy2C with its endogenous ligands, guanylin and uroguanylin, would enhance Ser40 phosphorylation in mouse striatal slices. See Supplementary Figure S1 for the blot-like images of each individual experiment.

**Fig. 8:**
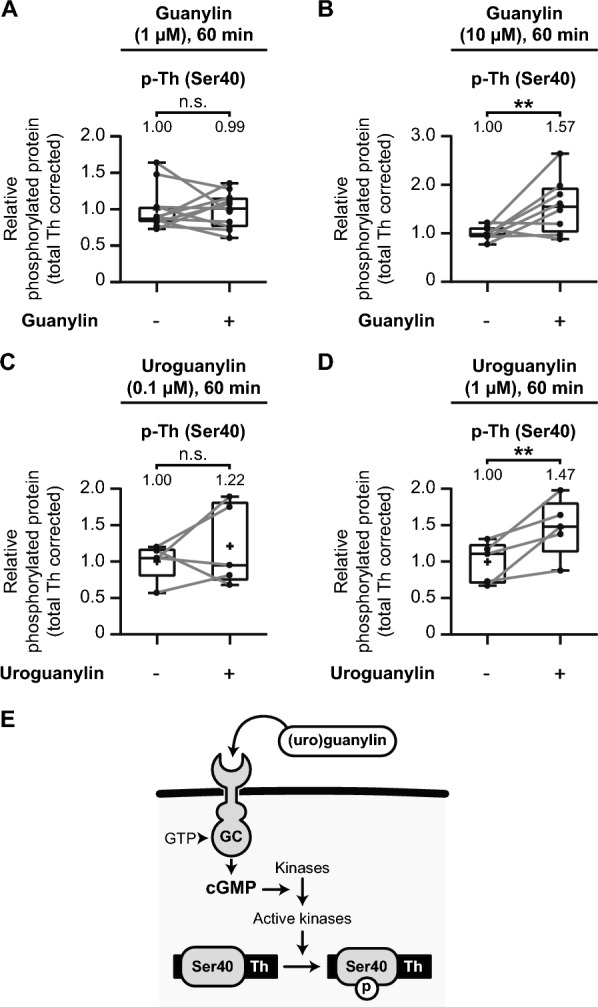
Gucy2C activation upregulates tyrosine hydroxylase Ser40 phosphorylation in the mouse striatum. Quantitative analysis of the effects of the effects of Gucy2C activation by guanylin and uroguanylin on tyrosine hydroxylase (Th) Ser40 phosphorylation in mouse striatal slices. **A**–**D** Exposure to the GUCY2C activators guanylin or uroguanylin for 60 min at various concentrations. **A** Guanylin (1 µM) has no significant effect on Th Ser40 phosphorylation (*n* = 11). **B** Guanylin (10 µM) significantly upregulates Th Ser40 phosphorylation (*n* = 8). **C** Uroguanylin (0.1 µM) does not significantly affect Th Ser40 phosphorylation (*n* = 5). **D** Uroguanylin (1 µM) significantly upregulates Th Ser40 phosphorylation (*n* = 5). **E** Schematic representation of the molecular mechanism whereby GUCY2C (GC) activation via guanylin and uroguanylin increases intracellular cyclic guanosine monophosphate (cGMP) levels. Elevated cGMP levels activate specific kinases, which in turn phosphorylate Th at Ser40, promoting dopamine biosynthesis

We incubated micro-dissected mouse striatal tissue with different concentrations of guanylin and uroguanylin, examining their effects on tyrosine hydroxylase Ser40 phosphorylation. First, exposure to 1 µM guanylin for 60 min did not significantly affect tyrosine hydroxylase Ser40 phosphorylation levels (Fig. 8A; *t*(10) = 0.09, *p* = 0.93, *M* = 0.99). However, increasing the concentration to 10 µM guanylin for 60 min resulted in a significant elevation in Ser40 phosphorylation (Fig. 8B; *t*(7) = 2.71, *p* = 0.03, *M* = 1.57), demonstrating that guanylin-mediated Gucy2C activation enhances tyrosine hydroxylase Ser40 phosphorylation in a dose-dependent manner.

Next, we explored the effects of uroguanylin, another endogenous GUCY2C ligand. Incubation with 0.1 µM uroguanylin for 60 min showed no significant increase in Ser40 phosphorylation (Fig. 8C; *t*(4) = 0.92, *p* = 0.41, *M* = 1.22). However, a higher concentration of 1 µM uroguanylin for 60 min significantly elevated tyrosine hydroxylase Ser40 phosphorylation levels (Fig. 8D; *t*(4) = 3.38, *p* = 0.03, *M* = 1.47), further reinforcing the dose-dependent relationship between Gucy2C activation and enhanced phosphorylation of Ser40.

Together, these results suggest that GUCY2C activation via its ligands, guanylin and uroguanylin, increases cGMP levels in the striatum, promoting tyrosine hydroxylase Ser40 phosphorylation (Fig. 8E). The upregulation of Ser40 phosphorylation through Gucy2C stimulation mirrors the effects observed with PDE inhibition, highlighting the crucial role of cyclic nucleotide signaling in modulating tyrosine hydroxylase activity.

### Phosphorylation of tyrosine hydroxylase at Ser40 has no adverse effect on total tyrosine hydroxylase levels

**Fig. 9:**
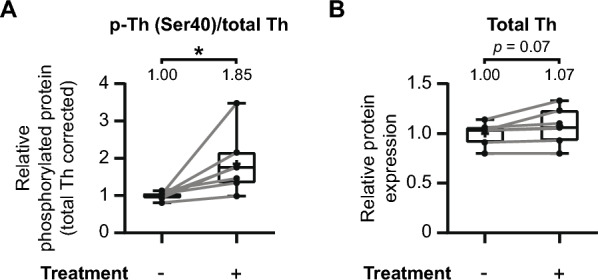
The phosphorylation of Ser40 has no adverse effect on overall tyrosine hydroxylase levels in the mouse striatum. The earlier discussed individual experiments that demonstrate significant increases in tyrosine hydroxylase Ser40 phosphorylation in the mouse striatum (Figs. 3, 7A, E, F, H, and I) were further examined on additional effects on overall tyrosine hydroxylase levels. **A** Quantitative analysis demonstrates a significant increase in relative Ser40 phosphorylation levels upon pharmacological treatment (*n* = 7). **B** These elevations in relative Ser40 phosphorylation are not accompanied by a discernable effect on overall tyrosine hydroxylase levels, although there is a trend (*p* = 0.07) towards a slight increase in tyrosine hydroxylase levels

Some studies have suggested that elevated phosphorylation of tyrosine hydroxylase at Ser40 might influence the degradation of tyrosine hydroxylase itself [101, 102]. To investigate whether such an effect is present, we examined the relationship between increased Ser40 phosphorylation and the overall levels of tyrosine hydroxylase (Fig. 9).

We assessed whether the treatments that enhanced Ser40 phosphorylation also affected the total tyrosine hydroxylase levels in experimental conditions (Fig. 3, 7A, E, F, H, and I). To facilitate comparisons across experiments, data were normalized to the average of the control condition for each animal. Area under the curve (AUC) values were then calculated for each condition per animal. As anticipated, treatments enhancing Ser40 phosphorylation led to a significant increase in relative Ser40 phosphorylation levels (Fig. 9A; *t*(6) = 2.89, *p* < 0.05, *M* = 1.85). However, this enhancement of Ser40 phosphorylation is not accompanied by a discernable effect on overall tyrosine hydroxylase levels (Fig. 9B; *t*(6) = 2.22, *p* = 0.07, *M* = 1.07), although demonstrating a trend towards a slight increase with none of the individual pairs demonstrating a negative effect. Consequently, it can be concluded that the phosphorylation of tyrosine hydroxylase at Ser40 has no adverse impact on the total levels of tyrosine hydroxylase.

### PDE inhibition and Gucy2C activation improve motor behavior in the 6-OHDA Parkinson’s disease mouse model

**Fig. 10:**
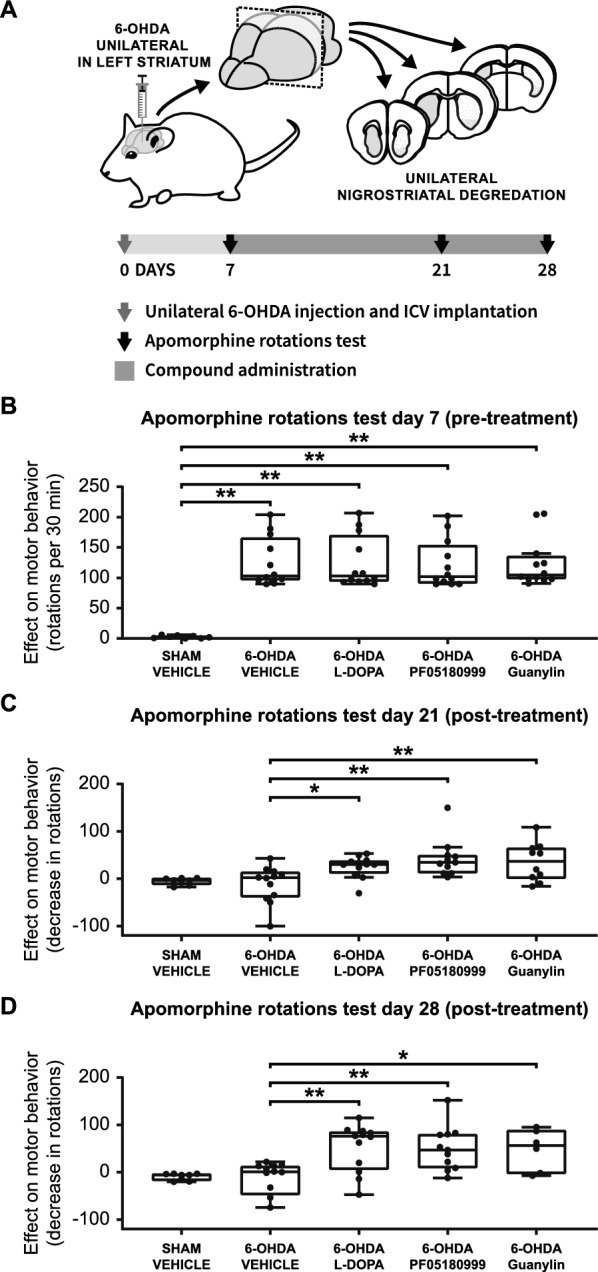
PDE inhibition and Gucy2C activation ameliorate impaired motor behavior in the 6-OHDA Parkinson’s disease mouse model. **A** Experimental Setup: Parkinson's disease model induction involved unilateral injection of 6-OHDA into the left striatum, causing unilateral degeneration of striatal dopaminergic terminals. On day 7, the first apomorphine rotations test assessed the efficacy of the lesions. Following the initial test, animals received three weeks of treatment with either vehicle, levodopa (L-DOPA), the PDE2A inhibitor (PF05180999), or the Gucy2C ligand (guanylin). Additional apomorphine-induced rotation tests were conducted after two and three weeks of treatment to evaluate the impact on motor behavior. Efficacy was measured by observing changes in rotation behavior, reflecting the effectiveness of the treatments. **B** Effect on motor behavior (rotations per 30 min) at day 7 following 6-OHDA induction (pre-treatment). All four 6-OHDA groups (*n* ≥ 11) exhibited significant increases in rotations compared to the sham group (*n* = 8), indicating the successful induction of motor deficits by 6-OHDA. **C** Effect on motor behavior (decrease in rotations compared to day 7 baseline) at day 21, following 2 weeks of treatment. Compared to 6-OHDA vehicle-treated animals (*n* = 12), L-DOPA (*n* = 12), PF05180999 (*n* = 11), and guanylin (*n* = 10) treatments demonstrate significant amelioration in motor complications. **D** Effect on motor behavior (decrease in rotations compared to day 7 baseline) at day 28, following 3 weeks of treatment. L-DOPA (*n* = 12), PF05180999 (*n* = 11), and guanylin (*n* = 6) treatments continue to show significant amelioration in motor complications compared to 6-OHDA vehicle-treated animals (*n* = 12)

Parkinson's disease is characterized by the loss of dopaminergic neurons, resulting in motor deficits [4, 103]. Here, we investigated the potential of PDE inhibition and Gucy2C activation as novel therapeutic strategies to alleviate these motor symptoms. To achieve this, we utilized the partial unilateral 6-hydroxydopamine (6-OHDA) mouse model (Fig. 10A), a well-established preclinical model for Parkinson's disease [104, 105]. This model involves surgically injecting 6-OHDA into the brain, leading to Parkinson's disease-like symptoms. To assess the efficacy of PDE inhibition and Gucy2C activation treatments, we employed the apomorphine rotations test, a valuable tool for the in vivo treatment efficacy in Parkinson's disease. We conducted a total of three rotation tests, with the initial test performed seven days after the 6-OHDA lesion and pre-treatment (Fig. 10B). Subsequent tests were conducted on day 21 (Fig. 10C) and day 28 (Fig. 10D) after the lesion, following two and three weeks of treatment, respectively.

Successful dopamine deafferentation was confirmed by baseline measurements pre-treatment, with all 6-OHDA-lesioned groups displaying motor deficits, as indicated by significant increases in unilateral rotation behavior (Fig. 10B; F(4, 51) = 17.69, *p* < 0.01). Apomorphine induced substantial changes in rotational behavior in the 6-OHDA-lesioned vehicle-treatment group (Fig. 10B; *t*(51) = 7.10, *p* < 0.01, *ΔM* = 122.7), the 6-OHDA-lesioned L-DOPA-treatment group (Fig. 10B; *t*(51) = 7.09, *p* < 0.01, *ΔM* = 122.5), the 6-OHDA-lesioned PF05180999-treatment group (Fig. 10B; *t*(51) = 6.92, *p* < 0.01, *ΔM* = 119.6), and the 6-OHDA-lesioned guanylin-treatment group (Fig. 10B; *t*(51) = 7.03, *p* < 0.01, *ΔM* = 121.5). However, after a two weeks treatment period, L-DOPA (Fig. 10C; *t*(48) = 2.72, *p* < 0.05, *ΔM* = 36.92), PF05180999 (Fig. 10C; *t*(48) = 3.93, *p* < 0.01, *ΔM* = 54.60), and guanylin (Fig. 10C; *t*(48) = 3.31, *p* < 0.01, *ΔM* = 47.22) significantly improved motor behavior. The improvement persisted after three weeks of treatment, for L-DOPA (Fig. 10D; *t*(44) = 3.87, *p* < 0.01, *ΔM* = 76.25), PF05180999 (Fig. 10D; *t*(44) = 3.69, *p* < 0.01, *ΔM* = 74.37), and guanylin (Fig. 10D; *t*(44) = 2.97, *p* = 0.015, *ΔM* = 71.58).

In conclusion, both PDE inhibition via the PDE2A inhibitor PF05180999 and Gucy2C activation via guanylin effectively alleviate motor complications induced by dopamine deafferentation in the 6-OHDA Parkinson's disease mouse model, to a similar extent as the gold standard treatment, L-DOPA. These results highlight the therapeutic potential of both PDE inhibition and Gucy2C activation in addressing the motor symptoms of Parkinson's disease.

## Discussion

In this study, we used an ex vivo pharmacological approach employing acute brain slices to investigate the phosphorylation of tyrosine hydroxylase, the rate-limiting enzyme in dopamine biosynthesis. The phosphorylation of its Ser40 plays a pivotal role in the regulation of tyrosine hydroxylase activity, thereby controlling endogenous dopamine production. Our findings confirm the pivotal role of cyclic nucleotide signaling in modulating Ser40 phosphorylation and extend this knowledge by exploring both PDE inhibition and GUCY2C activation as potential strategies to boost endogenous dopamine production via tyrosine hydroxylase Ser40 phosphorylation. These finding translated into improved motor behavior in a Parkinson's disease in vivo mouse model. Therefore, these results offer new avenues for research and potential therapeutic interventions in addressing the central feature of striatal dopamine deficiency in Parkinson's disease.

We initially reaffirmed that increasing cyclic nucleotide levels robustly upregulates Ser40 phosphorylation (Fig. 3). Ser40 phosphorylation boosts tyrosine hydroxylase activity and thereby enhances dopamine production [14, 25, 106]. Moreover, as in Parkinson’s disease nigrostriatal dopamine neurons progressively degrade [1, 107, 108], and some studies suggest that there is some sort of compensation for the extensive loss of these dopaminergic neurons via increased dopamine synthesis [68, 109–113], we investigated tyrosine hydroxylase levels and its manipulation via phosphorylation in a dopamine deficient situation. We used the Pitx3-deficiency mouse model [89–93] for selective loss of nigrostriatal dopamine neurons and, indeed, showed that the loss in dopaminergic neurons is accompanied by a proportional loss in tyrosine hydroxylase protein levels (Fig. 4). However, even in such a setting where a substantial proportion of dopaminergic neurons is lost, the relative Ser40 phosphorylation levels remain unchanged and are prone to manipulation in a cyclic nucleotide-dependent manner. Consequently, these data suggest that there are no major compensatory mechanisms upon nigrostriatal neuron degeneration that will give rise to changes in relative tyrosine hydroxylase Ser40 phosphorylation. In addition, our results are in support that nigrostriatal function predominantly adapts to the loss of nigrostriatal dopaminergic neurons in a more postsynaptic manner [107, 114–116] and supports that tyrosine hydroxylase regulation via cyclic nucleotide-mediated Ser40 phosphorylation can be an interesting approach to overcome striatal dopamine deficiency.

In fact, in a therapeutic perspective it is of crucial importance that the mechanism of tyrosine hydroxylase Ser40 phosphorylation is applicable as well in a setting where a substantial proportion of nigrostriatal dopamine neurons is lost. This in contrast to the administration of L-DOPA, the gold standard first-line treatment for Parkinson’s disease, as the progressive loss in nigrostriatal dopaminergic projections may actually be a factor that prevents the durability of L-DOPA therapy [13, 117–120]. Eventually, the ability to maintain physiological normal synaptic dopamine levels upon each L-DOPA dose will be lost, and thereby the ability to prevent overstimulation of postsynaptic dopamine receptors [13, 117, 121, 122]. Indeed, enhancing a more controlled way of dopamine synthesis that includes both the endogenous dopamine biosynthesis machinery and its underlying activating circuitry might prevent over-exploitation of the nigrostriatal postsynaptic signaling pathways when a substantial proportion of nigrostriatal dopamine neurons is lost. Therefore, boosting tyrosine hydroxylase Ser40 phosphorylation could provide a more durable alternative to the current treatments.

Furthermore, we demonstrate that enhanced tyrosine hydroxylase Ser40 phosphorylation has no effect on overall tyrosine hydroxylase levels (Fig. 9). This finding contrasts with the hypotheses put forth by some reports, suggesting that tyrosine hydroxylase phosphorylated at Ser40 is rapidly ubiquitinated for degradation [101, 102]. Indeed, the studies supporting this notion are either suggestive in nature [123, 124] or provide indirect evidence. Specifically, lactacystin [125, 126] or MG-132 [127–129] are used to demonstrate a link between enhanced Ser40 phosphorylation and proteasomal degradation. It's noteworthy that lactacystin and MG-132 are known to enhance intracellular cyclic nucleotide levels [130, 131], while cyclic nucleotide-stimulating agents themselves do not affect proteasome activity [132]. As confirmed in this study the connection between cyclic nucleotide signaling and enhanced Ser40 phosphorylation is well-documented [21, 26–31]. Therefore, this suggests that the observed link between enhanced Ser40 phosphorylation and proteasomal degradation in these in vitro studies may be indirect consequences of the experimental setup.

Moreover, our previous work demonstrated stable transient transfections and expression of a tyrosine hydroxylase variant with the Ser40 residue mimicking a stoichiometrically phosphorylated state [15]. This discovery contradicts the hypothesis that Ser40 phosphorylation leads to rapid tyrosine hydroxylase degradation. In support, our current study instead reveals a trend towards a subtle increase in tyrosine hydroxylase levels in the condition with enhanced Ser40 phosphorylation (Fig. 9B). This could be explained by the role of cAMP in positively regulating tyrosine hydroxylase mRNA expression and promoter activity [133–138], a pattern also observed in vivo in a Parkinson’s disease mouse model [139]. Therefore, the observed trend towards increased overall tyrosine hydroxylase levels in the context of cyclic nucleotide-mediated Ser40 phosphorylation could be attributed to elevated cyclic nucleotide levels. These observations collectively challenge the proposed relationship between Ser40 phosphorylation and tyrosine hydroxylase degradation.

Interestingly, our findings demonstrate that both PDE inhibition (Figs. 6 and 7) and GUCY2C activation (Fig. 8) significantly upregulate tyrosine hydroxylase Ser40 phosphorylation, an effect driven by elevated cyclic nucleotide levels (Fig. 5). This supports the role of cyclic nucleotide signaling as a critical modulator of endogenous dopamine production. By leveraging these two pharmacological strategies, we effectively can boost endogenous dopamine biosynthesis mechanisms, highlighting their potential as therapeutic tools in dopamine-deficient conditions such as Parkinson's disease.

Moreover, we observed that these findings translates into functional improvements in motor behavior (Fig. 10). Both PDE inhibition and GUCY2C activation ameliorated motor impairments in the 6-OHDA Parkinson’s disease mouse model, further underscoring their therapeutic relevance. The observed improvements in motor function suggest that targeting cyclic nucleotide pathways could represent a viable approach to managing Parkinson’s disease, especially given the underlying mechanism of Ser40 phosphorylation and its role in sustaining endogenous dopamine synthesis. These results are consistent with previous studies showing that cyclic nucleotide signaling positively impacts motor deficits in dopamine-deficient models [140–147]. Together, these findings offer compelling evidence that cyclic nucleotide-mediated signaling, whether through PDE inhibition or GUCY2C activation, holds promise as a novel therapeutic avenue for addressing striatal dopamine deficiency in neurodegenerative disorders such as Parkinson’s disease.

Notably, the beneficial effects of PDE inhibition and GUCY2C activation in the treatment of Parkinson’s disease extend beyond boosting the dopamine biosynthesis machinery, as it could provide a favorable disease-modifying role through multiple interrelated cyclic nucleotide-mediated signaling pathways that contribute to Parkinson’s disease pathophysiology [54, 61, 148]. In fact, cyclic nucleotide-mediated signaling appears to promote neuroprotective effects in various Parkinson’s disease models [141, 146, 147, 149–153]. Moreover, cAMP-mediated signaling promotes neuroprotective effects via the restoration of mitochondrial function [154–159], by attenuating α-synuclein cytotoxicity [156, 160–163], and via the downregulation of neuroinflammatory processes [150, 164–168]. In addition, cGMP-mediated signaling via GUCY2C signaling limits dopaminergic neuron vulnerability to toxic insults [61]. Therefore, cyclic nucleotide signaling can provide a way to alleviate the pathophysiological causes that are involved in the neurodegenerative processes fundamental to Parkinson’s disease [54]. As such, cyclic nucleotide-mediated signaling offers a dual approach in Parkinson's disease treatment, replenishing dopamine levels through tyrosine hydroxylase Ser40 phosphorylation to alleviate motor symptoms and concurrently promoting neuroprotection.

Our study highlights the therapeutic potential of modulating the endogenous dopamine biosynthesis pathway via tyrosine hydroxylase Ser40 phosphorylation as a strategy to ameliorate Parkinson’s disease symptomatology. Both PDE inhibition and GUCY2C activation effectively upregulate Ser40 phosphorylation, thereby boosting dopamine production, and are able to improve motor deficits in the 6-OHDA Parkinson's disease model.

While PDE inhibition offers a promising approach to boost cyclic nucleotide signaling and promote dopamine synthesis, it also presents challenges due to the widespread expression of PDEs across different brain regions, which could lead to off-target effects. Cyclic nucleotide-mediated signaling can play complex roles in various critical processes throughout the brain and body [34, 37, 46–49]. While this complexity makes them promising therapeutic targets, it also raises concerns about potential adverse effects. In fact, the abundance of specific PDEs in various (brain) regions limits the widespread therapeutic use of certain PDE inhibitors, as clearly demonstrated for example with PDE4 inhibitors [169–171]. Therefore, responses in off-target tissue activity could hamper the use of PDE inhibitors.

PDE2A, for example, is expressed both presynaptically and postsynaptically in dopaminergic systems [172], potentially complicating its therapeutic application by affecting postsynaptic signaling and introducing unintended consequences. Nonetheless, the availability of potent and specific inhibitors to PDE2A allowed us to investigate the effects of inhibiting a specific PDE in order to boost the endogenous dopamine biosynthesis pathway. Thereby, we successfully demonstrated that tyrosine hydroxylase Ser40 phosphorylation can be upregulated via PDE inhibition, proving the therapeutic potential of PDE inhibition to replenish striatal dopamine in situations where dopamine neurotransmission is affected, such as seen in Parkinson’s disease. Furthermore, the diversity in PDEs provides an opportunity to target specific PDEs that are less involved in non-dopaminergic brain regions [41, 46, 172], minimizing off-target effects. This ability to focus on more selective PDEs, or to employ region-specific delivery methods, could help mitigate these issues, offering a more tailored approach to dopamine regulation.

On the other hand, GUCY2C activation provides a more targeted mechanism for boosting dopamine synthesis than PDE2A, as this receptor is already enriched in midbrain dopaminergic neurons [60, 62, 63]. This receptor-specific strategy may circumvent some of the off-target concerns associated with targeting non-midbrain dopamine neuron specific/enriched PDE inhibition, allowing for a more direct modulation of tyrosine hydroxylase phosphorylation within the nigrostriatal pathway. Given the potential of GUCY2C activation to selectively boost dopamine production mechanisms in relevant brain regions, it represents a compelling strategy for targeted therapeutic intervention in Parkinson’s disease.

## Conclusion

Our findings suggest that enhancing cyclic nucleotide signalling—whether via PDE inhibition or GUCY2C activation—holds considerable promise for treating Parkinson’s disease by boosting endogenous dopamine production through tyrosine hydroxylase Ser40 phosphorylation. These non-invasive strategies could not only offer symptomatic relief but also neuroprotective benefits, addressing both dopamine depletion and neurodegenerative processes. Future research should focus on refining these approaches to maximize therapeutic efficacy while minimizing off-target effects, particularly by optimizing cyclic nucleotide modulation within the nigrostriatal dopaminergic circuitry. By doing so, it may be possible to develop a novel and targeted therapy that enhance the body’s own dopamine biosynthesis machinery, either as an adjunct to existing treatments like L-DOPA or as a stand-alone therapeutic strategy.

## Supplementary Information

Supplementary Material 1. Blot-like images corresponding to the minimum to maximum boxplots represented in figure 7 and 8. A Corresponds to Fig. 7A. B Corresponds to Fig. 7B. C Corresponds to Fig. 7C. D Corresponds to Fig. 7D. E Corresponds to Fig. 7E. F Corresponds to Fig. 7F. G Corresponds to Fig. 7Gand Fig. 7H. H Corresponds to Fig. 7I. I Corresponds to Fig. 8A. J Corresponds to Fig. 8B. K Corresponds to Fig. 8C. L Corresponds to Fig. 8D

## Data Availability

Data are available upon request from the corresponding author.

## Abbreviations

6-OHDA: 6-Hydroxydopamine
AC: Adenylyl cyclase
ATP: Adenosine triphosphate
AUC: Area under the curve
cAMP: Cyclic adenosine monophosphate
Cd: Caudal
cGMP: Cyclic guanosine monophosphate
CS: Corpus striatum
DMEM: Dulbecco’s Modified Eagle’s Medium
DMSO: Dimethyl sulfoxide
DTT: Dithiotretol
GC: Guanylyl cyclase
GFP: Green fluorescent protein
GTP: Guanosine triphosphate
GUCY2C: Guanylate cyclase-C
HiFBS: Heat inactivated fetal bovine serum
L-DOPA: L-3,4-dihydroxyphenylanine
PBS: Phosphate-buffered saline
PDE: Phosphodiesterases
PDL: Poly-d-lysine
PKA: CAMP-dependent protein kinase A
SEM: Standard error of the mean
Ser: Serine
Th: Tyrosine hydroxylase
