# Supplementary figures and images for: Phosphodiesterase inhibition and Gucy2C activation enhance tyrosine hydroxylase Ser40 phosphorylation and improve 6-hydroxydopamine-induced motor deficits
Source: Cell Biosci. 2024 Oct 25;14:132. doi: 10.1186/s13578-024-01312-7 (PMC11515495; doi:10.1186/s13578-024-01312-7)

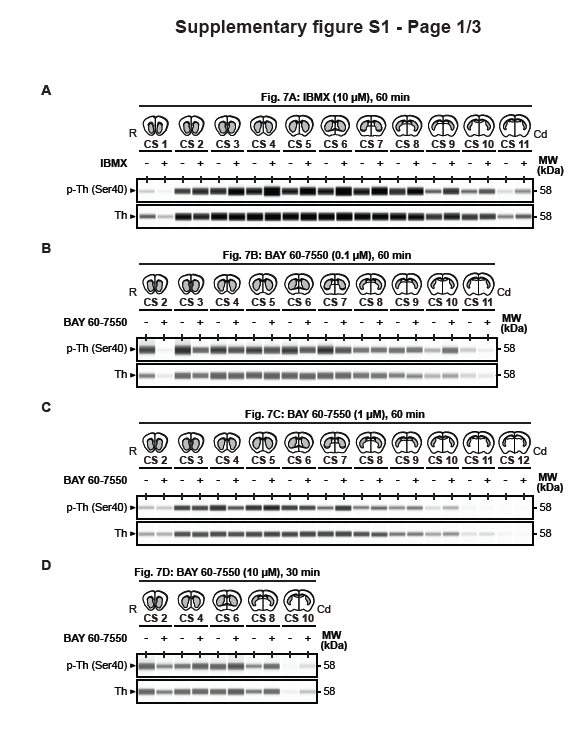


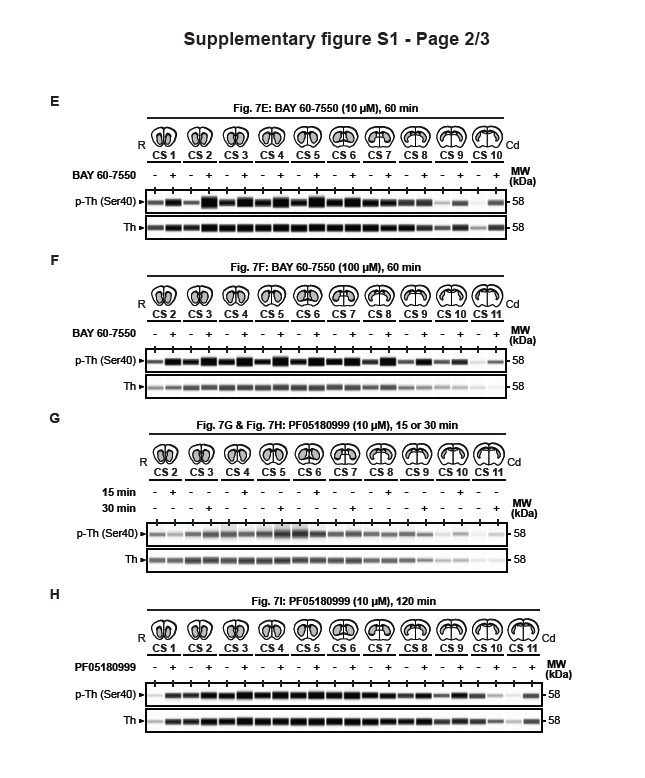


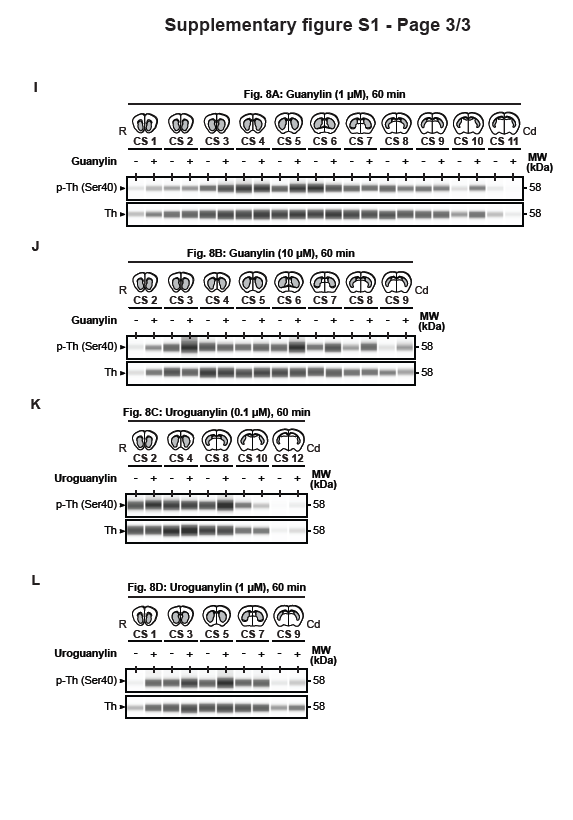

Supplement: Supplementary file 1 — Supplementary Material 1. Blot-like images corresponding to the minimum to maximum boxplots represented in figure 7 and 8. A Corresponds to Fig. 7A. B Corresponds to Fig. 7B. C Corresponds to Fig. 7C. D Corresponds to Fig. 7D. E Corresponds to Fig. 7E. F Corresponds to Fig. 7F. G Corresponds to Fig. 7Gand Fig. 7H. H Corresponds to Fig. 7I. I Corresponds to Fig. 8A. J Corresponds to Fig. 8B. K Corresponds to Fig. 8C. L Corresponds to Fig. 8D [file 13578_2024_1312_MOESM1_ESM.docx]
